# Selective Depletion of Adult GFAP-Expressing Tanycytes Leads to Hypogonadotropic Hypogonadism in Males

**DOI:** 10.3389/fendo.2022.869019

**Published:** 2022-03-16

**Authors:** Lucile Butruille, Martine Batailler, Marie-Line Cateau, Ariane Sharif, Valérie Leysen, Vincent Prévot, Pascal Vaudin, Delphine Pillon, Martine Migaud

**Affiliations:** ^1^ CNRS, IFCE, INRAE, Université de Tours, PRC, Nouzilly, France; ^2^ Univ. Lille, Inserm, CHU Lille, Laboratory of Development and Plasticity of the Neuroendocrine Brain, Lille Neurosciences & Cognition, UMR-S1172, Lille, France

**Keywords:** adult neural stem/progenitor cells, hypothalamus, glial fibrillary acidic protein, tanycytes, reproduction, GnRH, sexual behavior

## Abstract

In adult mammals, neural stem cells are localized in three neurogenic regions, the subventricular zone of the lateral ventricle (SVZ), the subgranular zone of the dentate gyrus of the hippocampus (SGZ) and the hypothalamus. In the SVZ and the SGZ, neural stem/progenitor cells (NSPCs) express the glial fibrillary acidic protein (GFAP) and selective depletion of these NSPCs drastically decreases cell proliferation *in vitro* and *in vivo*. In the hypothalamus, GFAP is expressed by α-tanycytes, which are specialized radial glia-like cells in the wall of the third ventricle also recognized as NSPCs. To explore the role of these hypothalamic GFAP-positive tanycytes, we used transgenic mice expressing herpes simplex virus thymidine kinase (HSV-Tk) under the control of the mouse *Gfap* promoter and a 4-week intracerebroventricular infusion of the antiviral agent ganciclovir (GCV) which kills dividing cells expressing Tk. While GCV significantly reduced the number and growth of hypothalamus-derived neurospheres from adult transgenic mice *in vitro*, it causes hypogonadotropic hypogonadism *in vivo.* The selective death of dividing tanycytes expressing GFAP indeed results in a marked decrease in testosterone levels and testicular weight, as well as vacuolization of the seminiferous tubules and loss of spermatogenesis. Additionally, GCV-treated GFAP-Tk mice show impaired sexual behavior, but no alteration in food intake or body weight. Our results also show that the selective depletion of GFAP-expressing tanycytes leads to a sharp decrease in the number of gonadotropin-releasing hormone (GnRH)-immunoreactive neurons and a blunted LH secretion. Overall, our data show that GFAP-expressing tanycytes play a central role in the regulation of male reproductive function.

## Introduction

In the mammalian brain, neurogenesis consists in the formation of new neurons from neural stem or progenitor cells (NSPCs). In adult, two discrete brain regions defined as neurogenic niches, namely the subventricular zone of the lateral ventricles (SVZ) and the subgranular zone of the hippocampal dentate gyrus (SGZ), retained this neurogenic potential. Within the specialized microenvironment of these niches, NSPCs exhibit a glial morphology with numerous processes and express astroglial markers such as the glial fibrillary acidic protein (GFAP) (1–5). *In vitro*, SVZ and SGZ GFAP-positive cells have the ability to generate floating spherical clusters called neurospheres, indicating their stemness potential (6). Moreover, genetic lineage tracing experiments have confirmed that the progeny of NSPCs derives from cells expressing GFAP (2). One strategy to elucidate the role of the NPSCs is to use the GFAP-Tk transgenic mouse line in which the herpes simplex virus (HSV) thymidine kinase (Tk) is expressed under the control of the Gfap promoter (GFAP-Tk). The antiviral agent ganciclovir (GCV) is a drug which, when phosphorylated by Tk, becomes a toxic metabolite that kills DNA-synthesizing cells. In the GFAP-Tk mouse model, only NSPCs are targeted and killed by GCV, without altering GFAP expression in other cell types such as astrocytes (2). When administered *in vitro* or *in vivo*, GCV therefore selectively eliminates dividing cells expressing both GFAP and Tk, leading to a strong decrease in SVZ and SGZ neurogenesis (2, 4, 7). Using this strategy, dividing GFAP-expressing cells have been shown to be the main population of progenitor cells responsible for constitutive neurogenesis in both neurogenic niches (2).

Recently, an additional discrete neurogenic niche located in the mediobasal hypothalamus [MBH; for reviews see (8) and (9)] has been documented in various species including human (10), mouse (11, 12), rat (13, 14), hamster (15, 16) and sheep (17–20). The hypothalamus, a diencephalic structure located around the 3^rd^ ventricle (3V), is involved in the control of critical physiological functions including food intake and reproduction. Within the hypothalamus, the arcuate nucleus (AN), the location of the central control of appetite and energy balance, contains orexigenic and anorexigenic neurons, including Neuropeptide Y (NPY) and Pro-opiomelanocortin (POMC) neurons, respectively. Besides these metabolic pathways, the hypothalamus also contains GnRH neurons, whose cell bodies and nerve endings are located respectively in the hypothalamic preoptic area (POA) and in the median eminence (ME) (21), stimulate the production by the pituitary gland of the gonadotrophic hormones, namely the luteinizing hormone (LH) and follicle-stimulating hormone (FSH). In turn, these gonadotrophins regulate gametogenesis and the production of sex steroids including testosterone and estradiol by the gonads (22).

In the MBH, radial glia-like cells lining the ventricular wall, namely tanycytes, have been identified as the endemic hypothalamic NSPCs that generate new neurons as well as glial cells in adult mice (23–25). Tanycytes, which have their cell body localized within the cellular layer lining the 3V wall and floor, send their unique process into the hypothalamic parenchyma. Four subpopulations of tanycytes can be distinguished based on their dorsoventral position, α1, α2, β1 and β2 tanycytes, from most dorsal to most ventral, down to the ME (26). All tanycyte subtypes express NSPC markers including the sex-determining region Y-box 2 (Sox2) (17, 24, 27), nestin (17, 28) and vimentin (17, 29, 30), but only the α1 subtypes and a restricted population of dorsal α2 subtypes express the *Gfap* transcript and the GFAP protein (25, 31, 32). In this study, we sought to investigate the role played by dividing GFAP-positive cells in physiological functions controlled by the hypothalamus using the GFAP-Tk transgenic mouse line.

## Materials and Methods

### Animals

All the experiments were approved by the Val de Loire animal experimentation ethics committee (CEEAVdL) and were in accordance with the Guidelines of the French Ministry of Agriculture and European regulations on animal experimentation (2010/63/EU). Experiments were performed in accordance with the local animal regulations (authorization N° 2015032613494293 of the French Ministry of Agriculture in accordance with the EEC directive). Experiments were performed on the transgenic mouse strain GFAP-Tk generated as described previously (33). Briefly, herpes-simplex virus thymidine kinase (HSV-Tk) is expressed under the control of the mouse *Gfap* promoter. Proliferating GFAP-positive cells expressing the transgene produce toxic nucleotide analogues in the presence of the antiviral agent GCV, which promote their apoptosis. Wild type (WT) and transgenic (Tg) male mice (2 months old) were obtained by mating transgenic females with non-transgenic males. Experiments were performed on four groups, the vehicle-treated WT mice (WT ctr), the GCV-treated WT mice (WT+GCV), the vehicle-treated Tg mice (Tg ctr) and the GCV-treated Tg mice (Tg+GCV).

### RNA Extraction and RT-PCR

Total RNA was extracted from the testes and striatum of three 2 months old mice using the RNeasy Mini Kit (Qiagen, Courtaboeuf, France) and was used for random-primed cDNA synthesis with SuperScript III reverse transcriptase (Thermo Fisher Scientific, Courtaboeuf, France) following the manufacturer’s instructions. Standard PCR was performed on cDNA aliquots using PlatiniumTaq (Thermo Fisher Scientific, Courtaboeuf, France) and the specific primers for the thymidine kinase T*k*; Forward primer, CGATGACTTACTGGCGGGTG; Reverse primer, GATACCGCACCGTATTGGCA) and Glyceraldehyde-3-phosphate dehydrogenase (*Gapdh*; Forward primer, CACCATCTTCCAGGAGCGAG; Reverse primer, GTTGAAGTCGCAGGAGACAAC) genes. PCR consisted of a first denaturing step at 94°C for 2 min, followed by 35 cycles of the following steps at 94°C for 30 sec, 55°C for 30 sec, and 72°C for 45 sec, ending with a 72°C extension step. PCR products were analysed on a 1.2% agarose gel containing 0.5 μg/mL ethidium bromide and visualized under a UV transilluminator.

### Neurosphere Cultures

Two WT and two Tg male mice (2 months old) *per* experiment (n = 3) were euthanized by cervical dislocation and the region containing the mediobasal hypothalamus was dissected out. Cells were mechanically dissociated using a cell scraper on a nylon membrane followed by an incubation for 7 min in TrypLE Express (Gibco 12604013, Life Technologies SAS, Courtaboeuf, France) and centrifuged 5 min at 300g. The resulting single-cell suspension was plated in a 25 ml flask in DMEM/F12 (Gibco, 21331-020, Life Technologies SAS, Courtaboeuf, France) supplemented with penicillin/streptomycin (PS), B27 and 20 ng/ml each of EGF (Invitrogen, 53003-018, Invitrogen) and bFGF (Invitrogen, 13256-029, Life Technologies SAS, Courtaboeuf, France). Cultures were incubated in humidified chambers with 5% CO_2_ at 37°C. Since neurospheres are composed of a heterogeneous mixture of neural stem cells (NSCs) and neural progenitors, it is crucial to enrich the culture with NSCs. To expand specifically the pool of NSCs at the expense of neural progenitors that have more limited self-renewal potential neurospheres can be serially passaged (34). Since progenitors are eliminated during the first passages, tertiary neurospheres are considered to be mostly composed of NSCs, and were used in this study. After 7 days *in vitro*, primary neurospheres underwent a first passage and were centrifuged for 5 min at 1000 rpm and incubated for 7 min in Accutase (Gibco A11105-01, Life Technologies SAS, Courtaboeuf, France). A mechanical dissociation into single-cell suspension using a pipet tip was performed, then the cells were seeded at a density of 10,000 cells/ml to obtain secondary neurospheres. The same protocol was used to culture tertiary neurospheres. To examine the effect of GCV on neurosphere formation, tertiary neurospheres of WT and Tg mice were cultured in the presence or absence of GCV (10 µM final concentration, Sigma Aldrich, G2536, St. Quentin Fallavier, France) for 2 weeks. During this long-term culture, the medium was supplemented every two days with growth factors. At the end of the 2 week-culture period, neurospheres were counted and their size measured for all the conditions.

### *In Vivo* GCV Administration

Two months old WT ctr (n=9), WT+GCV (n=8), Tg ctr (n=9) and Tg+GCV (n=11) male mice were housed in pairs and the pairs were separated by a Plexiglas partition to maintain visual and olfactory contact. GCV was administrated by intracerebroventricular infusion at a rate of 0.11 µl/hr for 28 days using an osmotic micropump (Model 1004, Alzet, Charles River, Saint-Germain-Nuelles, France). Micropumps connected to cannula (Brain Infusion Kit 2, Alzet, Charles River, Saint-Germain-Nuelles, France) were filled with 2 mM GCV or with saline solution and primed for 48 hours at 37°C. For implantation, mice were anesthetized with 100 mg/kg ketamine and 10 mg/kg xylazine and fixed to a stereotaxic frame after loss of reflexes. During surgery, the eyes were protected with ocry-gel and a local anesthetic (procaine) was injected under the skin of the skull. A micropump was introduced subcutaneously and the cannula was implanted into the 3V at 1.7 mm posterior to the Bregma and at a depth of 5 mm (Paxinos atlas). Following surgery, all mice were injected with 0.1 mg/kg morphine analgesic (0.3 mg/ml Buprecare) and 6 ml/kg injectable antibiotic (10 mg/kg Depocilline).

#### Food Intake and Body Weight Assessment

Body weights and food intake were measured weekly for 5 weeks starting one week before cannulation at week 0 (W0) until the end of the experiment at week 4 (W4), with week 1 (W1) corresponding to the surgery. To get accurate food intake measurement, mice were housed individually while social interactions (odors, vocalization and sight) were maintained with holes in the cage separator. Food intake was measured by giving a weighted amount of food and measurement of the left over/refusals on a weekly basis (35).

#### Behavioral Analysis

All the behavioral tests took place in the last week of treatment (W4).

##### Sexual Behavior

All sexual behavioral experiments were performed during the dark phase of the dark/light cycle, 1 hour after lights off. Tests were filmed with an infra-red light in a dark room. Two weeks before W0, male mice were placed for a week with a receptive female (in oestrus phase) to gain sexual experience. At W4, males were tested in an open field (50 x 50 cm) with a receptive female for 30 minutes. Females were ovariectomized and implanted with Silastic implants (Dow Corning, Saint Denis, France) containing 50 μg of E2-benzoate (Sigma-Aldrich). Four hours before the tests, females were given a subcutaneous injection of 1 mg of progesterone (Sigma-Aldrich, P6149, St. Quentin Fallavier, France) diluted in 100 μL of oil to induce receptivity (36). The latencies and frequencies of mounts and intromissions were recorded. The sexual preference of males was also evaluated. Males were placed in the centre of a three-compartment chamber separated by Plexiglas with an opening at the base permitting olfactory and visual contact. Following a 10 min period of habituation, a receptive female (detected by a vaginal smear) and an unfamiliar male were each placed in one of the side compartments of the chamber. The time spent by the tested male mouse near each compartment was recorded for 10 min.

##### Anxiety Levels

Anxiety level in the four groups was evaluated using the elevated plus maze and the marble burying tests (37). The elevated plus maze consists of two closed and two open cross-shaped arms (5 cm wide x 40 cm long) elevated 50 cm from the floor. Male mice were placed in the centre of the device and were allowed to explore the arms for 5 minutes. The number of entries and the time spent in each area of the device were recorded, i.e. the closed arms, the open arms, the distal zones of the open arms and the centre of the device.

In the marble burying test, male mice were placed in a test cage (15 cm wide x 33 cm long) containing fresh bedding. Twenty marbles were distributed evenly in the cage in 5 rows and a lid was placed on top of the cage. Animals were left undisturbed for 15 minutes, after which the number of marbles buried was recorded. A marble was counted as being buried if at least 2/3 of it had been covered by bedding.

#### Tissue Preparation

At W5, animals were anesthetized, blood was collected from the abdominal artery and serum was frozen for posterior hormone quantification. Following intracardial perfusion with 4% paraformaldehyde, brains were collected and after 24 h post-fixation in 4% paraformaldehyde, were cryoprotected in 30% sucrose at 4°C.

Testes, seminal vesicles, preputial glands and intestine (ileum) were dissected out and weighted. Testes and intestine samples were fixed by immersion in Bouin’s fixative solution for 48 hours, embedded in paraffin, then cut in 9 µm thick sections and stained with haematoxylin and eosin for histology. The densities of spermatogonia and spermatocytes per seminiferous tubule (number of cells per mm²) were assessed. Three images per mouse (2-3 mice per group) and 3 seminiferous tubules per image were used for this quantification.

#### Immunohistochemistry

Brain coronal sections (25 μm thick) cut from the anterior POA in a caudal direction until the premamillary recess, were collected using a cryostat (Leica CM 3050 S). The sections were directly mounted on Superfrost Plus slides (Fisher Scientific, Illkirch, France) and stored at -80°C until used for immuno-histochemistry. For all antibodies (**Table 1**), normal serum IgGs of appropriate species were used as negative controls. For each mouse five sections separated by 100 µm and 160 µm at different rostro-caudal levels of the POA and the MBH respectively were used for immunohistochemistry. To simultaneously permeabilize and block nonspecific binding sites, sections were placed in a solution of 5% normal horse serum and 0.3% Triton X-100 in TBS (TBSTH) for 30 min and incubated in the same buffer containing the primary antibodies (**Table 1**). They were then incubated with secondary antibodies (**Table 1**) and mounted in Fluoromount (SouthernBiotech, Birmingham, AL, USA) for observation.

**Table 1:** Primary and secondary antibodies used for immunohistochemistry.

Primary antibody	Manufacturer, species type, cat. no.	Unmasking step	Dilution	Secondary antibody	Manufacturer, cat. no.	Dilution
GFAP	Dako, rabbit polyclonal, #Z0334		1,1000	Donkey anti-rabbit IgG, Alexa 488	Molecular Probes, #A21206	1,600
Sox2	R&D systems, goat polyclonal, # abAF2018	Sodium borohydrure 0.1%	1,300	Donkey anti-goat IgG, Alexa 555	Molecular Probes, #A21432	1,600
Vimentin	Millipore, chicken polyclonal, #AB5733		1,2000	Donkey anti-chicken IgY, Fluorescein isothiocyanate	Jackson ImmunoResearch #703095155	1,600
ERα	Santa Cruz-MC20, rabbit polyclonal, #sc542		1,200	Donkey anti-rabbit IgG, Alexa 488	Molecular Probes, #A21206	1,600
GnRH	Polyclonal rabbit (no. 19900)		1,10000	Donkey anti-rabbit IgG, Alexa 488	Molecular Probes, #A21206	1,600
Kisspeptin	Polyclonal rabbit (no. 564)	Citric acid (pH 6)	1,5000	Donkey anti-rabbit IgG, Alexa 555	Molecular Probes, #A31572	1,600
POMC	Phoenix, rabbit polyclonal, #H02930	Sodium borohydrure 0.1%	1,400	Donkey anti-rabbit IgG, Alexa 488	Molecular Probes, #A21206	1,600
NPY	Sigma, rabbit polyclonal, #N9528	Citric acid (pH 6)	1,10000	Donkey anti-rabbit IgG, Alexa 488	Molecular Probes, #A21206	1,600

#### Quantification of Immunolabelled Cells

The quantification of immune-positive cells was performed using a computerized image analysis system, Mercator (Explora Nova, La Rochelle, France), consisting of a microscope (Zeiss Axioscop) equipped with a motorized stage, a fluorescent lamp and a CDD video camera. Under a magnification of 10X, three hypothalamic anatomical regions of interest (ROI), the median eminence (ME), the arcuate nucleus (AN) and the ventromedial hypothalamus (VMH, except the ventricular border) were determined (25). Two methods, manual and automatic, of quantification were used. The number of GFAP-positive tanycytic cell bodies lining the ventricular wall was manually quantified in WT ctr and Tg+GCV male mice. For ERα and the cell bodies of the GnRH neurons in the POA the low density, and the nature of the labeling, respectively nuclear and cytoplasmic, allowed manual counting (number of cells/mm²). In the VMH, Sox2+ cells located in the hypothalamic parenchyma are dispersed also allowing manual quantification (number of cells/mm²). In the AN and ME, the ventricular wall consists entirely of Sox2+ cells (for control groups) stucked together hence difficult to isolate, making manual counting impossible, so an automatic quantification was used as previously described (38). Briefly, a gray level threshold was established to detect the immunolabelled area considered positive when the intensity of labeling was greater than the threshold. The labeling area corresponded to the intensity of pixels in the ROI relative to the surface of the ROI. To avoid any experimental bias, the section labeling of the four groups of mice was performed in parallel. Likewise, for each marker, identical image acquisition parameters and exposure times were used.

#### Image Capture for Immunohistochemistry

All images (1,024 x 1,024 pixels) for figure preparation were acquired using a confocal microscope (Zeiss, LSM 700, objective 40X) with Zen software (Carl Zeiss, Oberkochen, Germany). Images shown in the figures were pseudo-coloured using LSM Image Browser software (Carl Zeiss, Thornwood, NY), and Photoshop (Adobe Systems, San Jose, CA) was used on the resulting tiff files only to adjust for brightness and contrast.

#### Hormone Level Measurements

Two hours before euthanasia, males were injected intraperitoneally with 15 IU of human chorionic gonadotropin (hCG) (Intervet, France) diluted in physiological serum so that testosterone contained in mouse testes is fully secreted (36). Plasma testosterone concentrations were assayed using a RIA (radioactive immunoassay) using ^3^H-testosterone as previously described (36). The sensitivity of the assay was 0.06 ng/mL and the intra-assay coefficient of variation was 8.5%.

Plasma cortisol concentrations were measured using a direct radio-immunoassay method as previously described (39). The sensitivity of the assay was 0.25 ng/mL and the intra-assay coefficient of variation was 8.6%.

Serum FSH levels were measured using a commercial ELISA kit (Endocrine Technologies, Inc., ERKR7014) following manufacturer’s instructions. The sensitivity was 0.05 ng/ml and the intra-assay coefficient of variation was 3.42%.

Serum LH levels were measured using a sensitive sandwich ELISA (40). The sensitivity was 0.25 ng/ml and the intra-assay coefficient of variation was 7.73%.

### Statistical Analysis

Statistical analyses were performed with GraphPad Prism5 software (GraphPad Software, San Diego, CA). Data were compared using a non-parametric Kruskal-Wallis test followed by a Dunn’s post-test. A one-way ANOVA followed by a Bonferroni post-test was used to compare the size of the neurospheres after GCV treatment. A two-way ANOVA was used to compare body weights and food intake, and to compare the number of entries and the time spent in each area of the elevated plus maze. A Wilcoxon test was used to compare the sexual preference and the attractiveness of males. Differences were considered statistically significant for a p-value < 0.05. Data presented in the histograms are mean values ± standard error of the mean (S.E.M.).

## Results

### GFAP-Expressing Cells Are the Main Source of Hypothalamic Proliferative Cells *In Vitro*

**Figure 1:**
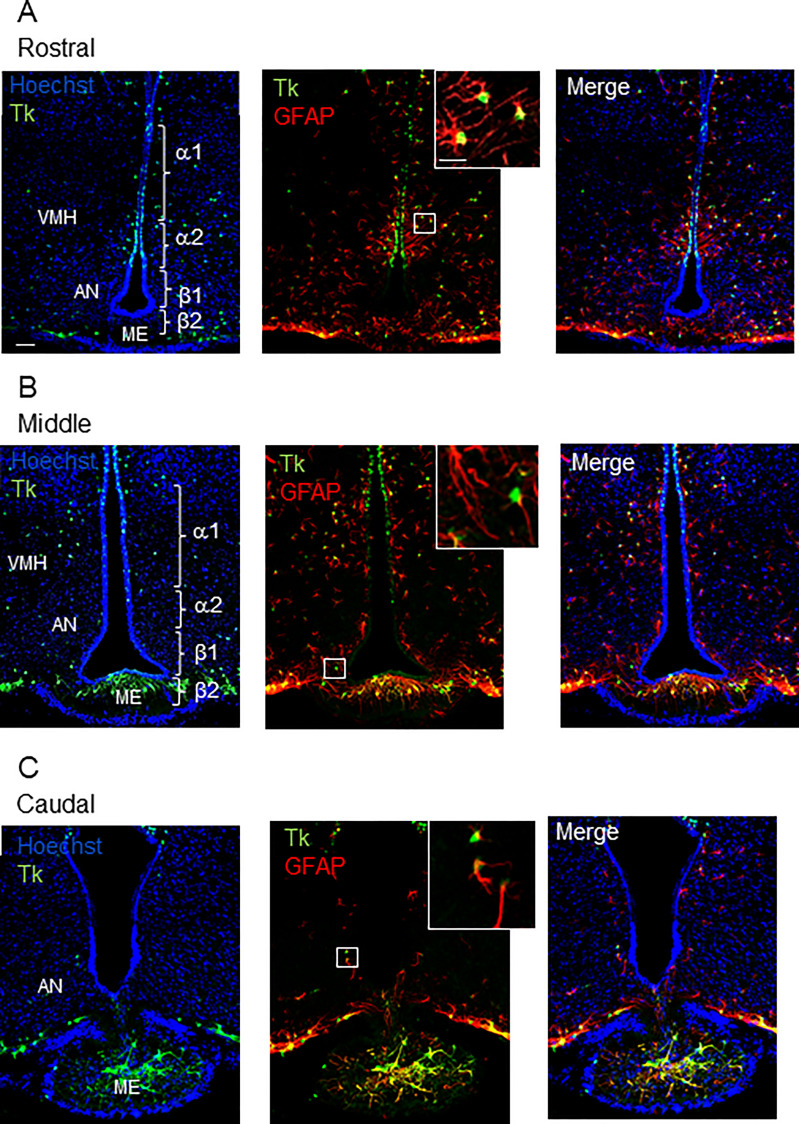
Distribution of GFAP-positive/Tk-positive cells in the MBH of GFAP-Tk male mice. Rostral **(A)**, middle **(B)** and caudal **(C)** MBH showing for each panel, from left to right and up to down, confocal images of nuclei coloration with Hoechst (blue), Tk (green), GFAP (red), Hoechst/Tk, Hoechst/GFAP expression and the merge image. Insets are high magnification showing Tk (green), and GFAP (red) immunopositive cells. All the Tk-positive cells are located in the ependymal layer of the VMH and the dorsal AN, corresponding to the localization of α1 and a subset of α2 tanycyte subtypes. GFAP-positive/Tk-positive cells are also detected in the AN and ME parenchyma. VMH, ventro-medial hypothalamus; AN, arcuate nucleus; ME, median eminence; Tk, thymidine kinase. Scale bars, 20 µm; Insets: 5 µm.

In the Tg mice model in which the herpes thymidine kinase is expressed under the control of the *Gfap* promoter (GFAP-Tk), double-labelling was used to assess the overlapping expression of GFAP and HSV-Tk at the single-cell level. Confocal analysis of cells showed that 100% of the Tk-positive cells also expressed the astroglial marker GFAP regardless of the rostro-caudal level of the hypothalamus (**Figures 1A–C**). No Tk-positive/GFAP-negative cells were found and only very few GFAP-positive cells with no detectable level of Tk could be observed in the ependymal layer of the 3V. High level of GFAP labeling could be observed within the ME, especially at the caudal level (**Figure 1C**). These results confirm the limited expression of Tk in the GFAP-positive cells in the hypothalamus as previously reported in the SVZ and SGZ (2).

Confocal image analysis showed that Tk-positive/GFAP-positive cells lay in the ependymal layer of the MBH corresponding more specifically to the α-tanycyte subtypes (**Figure 1A**) and in the MBH parenchyma corresponding to parenchymal progenitors (**Figures 1A, B**). A few Tk-positive cells were also observed in the ME (**Figures 1B, C**). Regarding their anatomical localization, ventricular Tk-positive/GFAP-positive cells will be referred to as GFAP-positive tanycytes thereafter.

**Figure 2:**
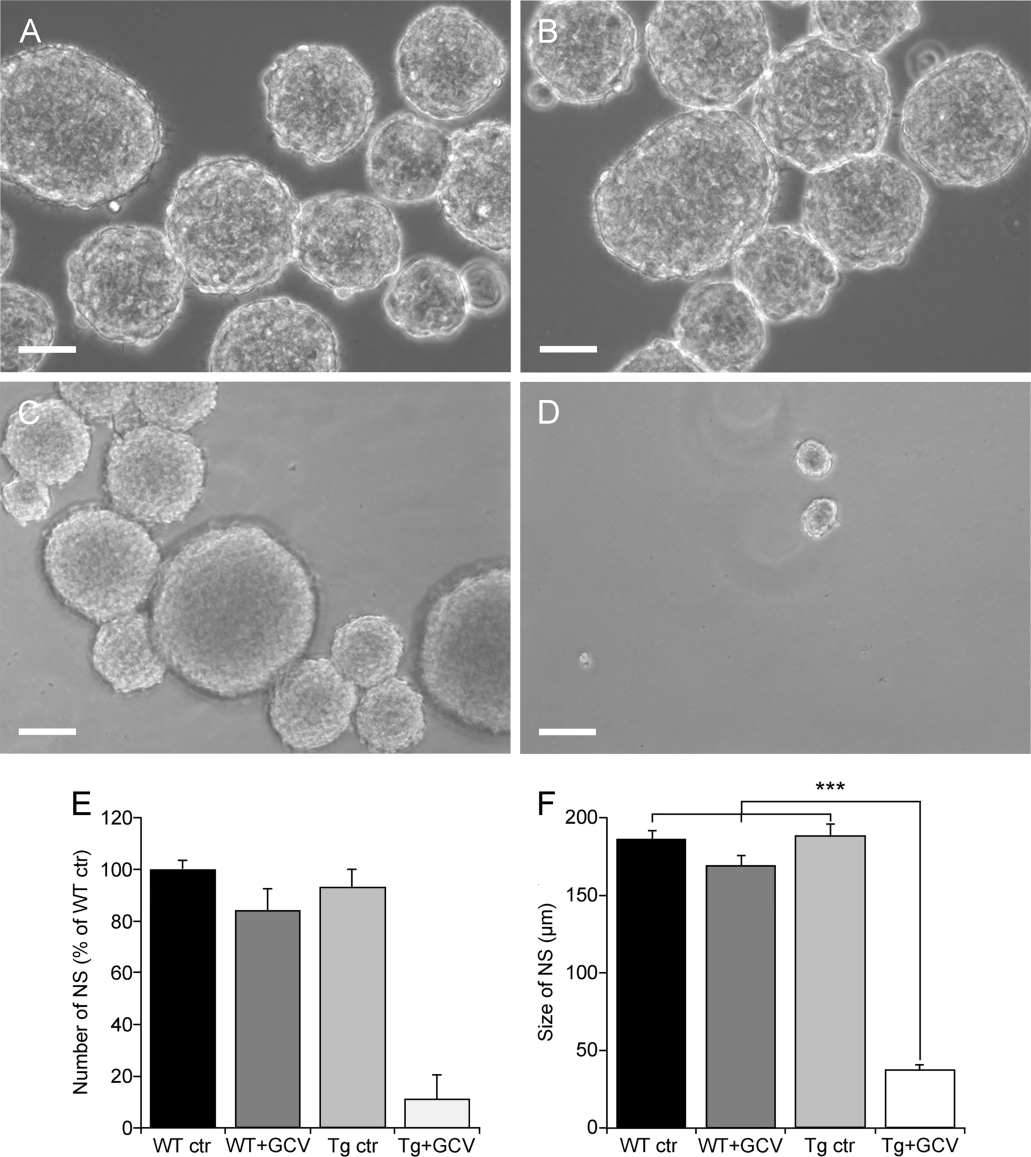
GFAP-expressing cells are the predominant source of proliferative cells within the hypothalamus. Representative images of floating hypothalamic tertiary neurospheres (NS) derived from wild type mice (WT ctr; **A**), wild type mice receiving a ganciclovir treatment (WT+GCV; **B**), GFAP-Tk Tg mice (Tg ctr; **C**) and GFAP-Tk Tg mice treated with an i.c.v. ganciclovir injection (Tg+ GCV; **D**). Scale bar, 100 µm. Number **(E)** and size **(F)** of hypothalamic neurospheres after a 2-week culture period in presence of GCV. Data are expressed as the mean ± SEM, WT n=2 and Tg mice n>3 for each group, n=3 experiments. ***p<0.001.

To determine the contribution of dividing GFAP-expressing cells to NSPCs isolated from hypothalamic tissue, we used the neurosphere assay, which is the most appropriate *in vitro* assay for detecting the presence of NSCs (41). We therefore compared the *in vitro* self-renewal potential of dividing hypothalamic cells. To specifically expand the pool of NSCs at the expense of progenitors, neurospheres were serially cultured to obtain tertiary neurospheres, so as to eliminate progenitors. The number of tertiary neurospheres, composed only of NSCs, was comparable between non-transgenic mice (WT ctr), GCV-treated non-transgenic mice (WT+GCV) and saline-treated transgenic mice (Tg ctr; **Figures 2A-C** respectively). In contrast, GCV almost completely prevented neurosphere formation from adult hypothalamic tissue derived from transgenic mice (Tg+GCV; **Figure 2D**). GCV treatment reduced the number of neurospheres from Tg+GCV mice by 89.2 ± 9.5% (**Figure 2E**). Furthermore, the rare neurospheres produced from Tg male mice treated with GCV were significantly smaller than those from control mice (**Figure 2F**; Bonferroni multiple comparison, p<0.0001). These results show that the ability of adult hypothalamic tissue to produce neurospheres *in vitro* was significantly reduced after transgenic-targeted elimination of dividing GFAP-positive cells, indicating that these GFAP+ cells contribute a very large part to the pool of hypothalamic NSPCs.

### *In Vivo* Central GCV Administration in Tg Mice Modified the Expression of NSPC Markers in the MBH

To determine whether GFAP-expressing tanycytes could constitute the major pool of NSPCs in the adult hypothalamus, two months old WT and Tg mice were subjected to 4-week saline and GCV infusion in the 3V using stereotaxically implanted cannulas and osmotic minipumps (WT ctr; WT+GCV; Tg ctr; Tg+GCV).

**Figure 3:**
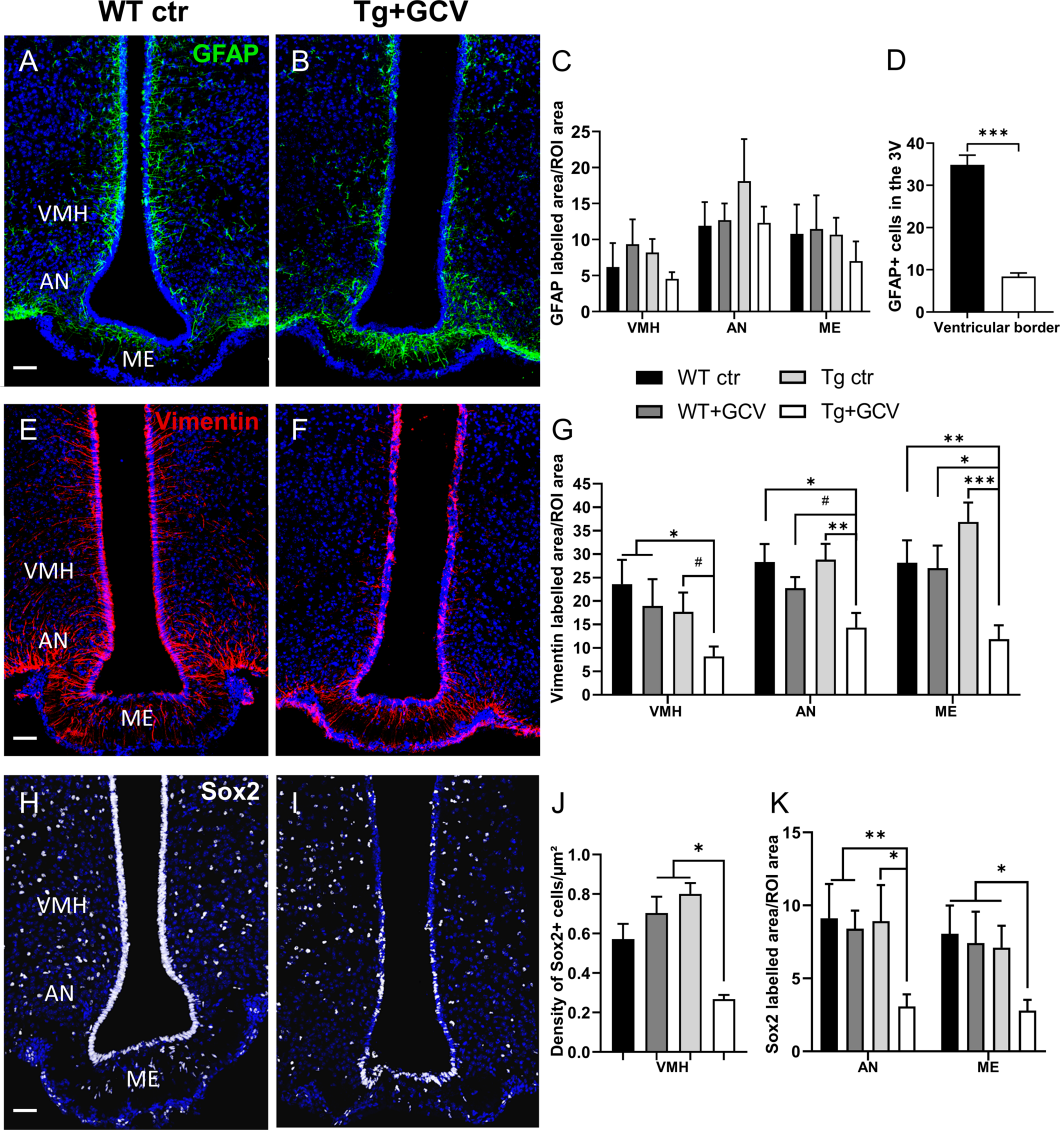
In vivo central GCV administration in Tg mice impaired expression of NSPC markers within the MBH. Representative images of GFAP expression measured in the mediobasal hypothalamus of control **(A)** and Tg+GCV male mice **(B)**. Statistical analysis revealed no variation of GFAP expression in the VMH, AN and ME **(C)**. In contrast, the number of GFAP-positive tanycytes located in the ventricular border is statistically lower in Tg+GCV male mice as compared to control mice **(D)**. Confocal images of Vimentin expression in the mediobasal hypothalamus of control **(E)** and Tg+GCV male mice **(F)**. Tg+GCV male mice display a significantly decreased Vimentin expression in the VMH, NA and ME **(G)**. Representative images of Sox2 expression in the mediobasal hypothalamus of control **(H)** and Tg+GCV male mice **(I)**. Statistical analysis revealed significantly less Sox2 positive cells in the ventromedial hypothalamus (VMH) **(J)**, in the AN and in the ME **(K)** in Tg+GCV male mice. Data are expressed as the mean ± SEM, n≥5 for each group. *p<0.05, **p<0.01. ***p<0.001, ^#^p=0.06 or 0.07. Scale bars, 50 µm.

In order to assess the effect of the GCV treatment on the expression of NSPC markers, brain slices were cut and immunohistochemical analyses were performed. The expression of NSPC markers, including GFAP (**Figures 3A–D** and **Supplementary Figures S1A, B**), vimentin (**Figures 3E–G** and **Supplementary Figures S1C, D**) and Sox2 (**Figures 3H–K** and **Supplementary Figures S1E, F**) was quantified using immunolabelling techniques. In Tg mice, the GCV treatment had no effect on the GFAP labelling in the parenchyma of the VMH (Kruskal-Wallis, p>0.05), AN (Kruskal-Wallis, p>0.05) or ME (Kruskal-Wallis, p>0.05) globally, when compared with the three control groups (**Figure 3C**). Although GFAP labelling remains unchanged in the MBH after GCV treatment, a detailed analysis of the ventricular border revealed that the number of GFAP-positive tanycytic cell bodies is reduced in Tg+GCV mice when compared to WT ctr littermates (Mann-Whitney, p=0.0002; **Figure 3D**). These results suggest that *i.c.v*. GCV treatment selectively promotes the depletion of GFAP-positive tanycytes along the wall of the 3V. The expression of vimentin (**Figures 3E, F**), a type III intermediate filament protein expressed in neural stem cells, was significantly reduced in the VMH, AN and ME of the Tg+GCV group as compared to the control groups (VMH: Kruskal-Wallis, p=0.05; Dunn’s, p<0.05; AN: Kruskal-Wallis, p=0.03; Dunn’s, 0.008<p<0.07; ME: Kruskal-Wallis, p=0.003; Dunn’s, 0.0004<p<0.01 **Figure 3G**). These results suggest that the ablation of dividing GFAP-positive tanycytes induces depletion in the α-tanycyte population in the wall of the 3V, but may also lead to a decrease in the β-tanycyte population forming the floor of the 3V. In addition to a marked depletion of Sox2-positive tanycytes (**Figures 3H, I**), the density of Sox2-positive cells in the parenchyma of the VMH, was significantly lower in the Tg+GCV group than in the WT+GCV and Tg ctr groups (Kruskal-Wallis, p=0.01; Dunn’s, p=0.04 and p=0.05 respectively **Figure 3J**). A significant decrease in Sox2-positive cell density was also observed in the AN (Kruskal-Wallis, p=0.02; Dunn’s, 0.005< p<0.03) and the ME (Kruskal-Wallis, p=0.04; Dunn’s, 0.02<p<0.04; **Figure 3K**). Altogether, these results support the view that i) GCV treatment resulted in NSPCs depletion in the MBH and ii) GFAP-expressing tanycyte ablation could lead to depletion of all tanycytic populations.

### *In Vivo* Depletion of Hypothalamic GFAP-Positive Tanycytes Did Not Affect Body Weight and Food Intake

As adult hypothalamic neurogenesis has been associated with the control of food intake and metabolism (12, 23, 42, 43), animals and their food intake were therefore weighed daily from week 0 (W0) to week 4 (W4) after GFAP-positive tanycyte depletion. A two-way ANOVA demonstrated that the GCV administration did not modify the body weight or food intake of the Tg-GCV mice when compared to the three control groups of mice (p>0.05, **Supplementary Figure S2A**, and p>0.05, **Supplementary Figure 2B**, respectively). The balance between anorexigenic neurons expressing Pro-opiomelanocortin (POMC) and orexigenic neurons expressing neuropeptide Y (NPY) mainly mediates metabolic activity including feeding and body weight regulation. In transgenic GCV-treated mice, the number of neurons immunolabelled for POMC (WT ctr: 37.78 ± 8.45; WT+GCV: 32.06 ± 9.31; Tg ctr: 45.84 ± 10.9; Tg+GCV: 30.91 ± 8.08; Kruskal-Wallis, p>0.05; **Supplementary Figure 2C**) and NPY (WT ctr: 10.45 ± 1.81; WT+GCV: 8.97 ± 0.43; Tg ctr: 13.96 ± 1.3; Tg+GCV: 13.72 ± 2.43; Kruskal-Wallis, p>0.05; **Supplementary Figure 2D**) did not differ from the control groups. Taken together these data indicate that ablation of hypothalamic GFAP-positive tanycytes did not cause any marked alteration in the regulation of body weight or food intake during the four weeks of treatment.

### Depletion of Hypothalamic GFAP-Positive Tanycytes Causes Hypogonadotropic Hypogonadism

**Figure 4:**
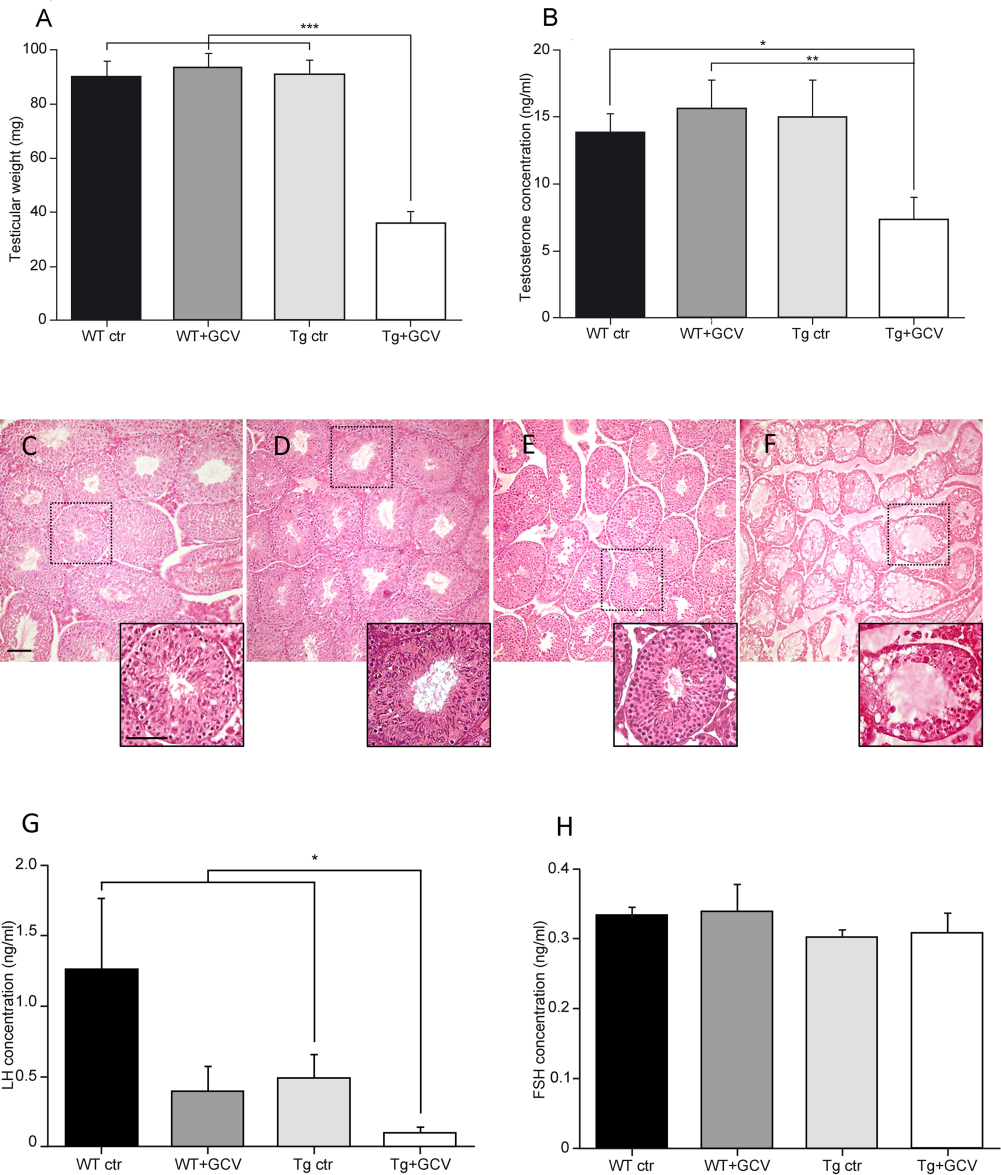
Alteration of gonadal functions after central GCV administration in Tg mice. Tg+GCV male mice display decreased testicular weight **(A)** and plasma testosterone concentrations after hCG (human chorionic gonadotropin) injection **(B)**. Testicular histology of WT ctr **(C)**, WT+GCV **(D)**, Tg ctr **(E)** and Tg+GCV **(F)** male mice revealed alterations and vacuolization of seminiferous tubules after central GCV administration in Tg mice, scale bars, 50 µm Insets show a higher magnification image of a single seminiferous tube for each group of mice. Scale bar, 200 µm. Tg+GCV male mice display decreased serum LH concentration **(G)** while serum FSH concentration is not affected by the treatment **(H)**. Data are expressed as the mean ± SEM, n≥3 for each group. *p<0.05, **p<0.01, ***p<0.001.

We next investigated the role of adult GFAP-expressing tanycytes on reproduction, a major neuroendocrine function controlled by the hypothalamus. The effect of 4-week GCV treatment on the weight of testes, seminal and preputial glands was examined. Tg+GCV mice showed a stricking decrease in testes weight (Kruskal-Wallis, p<0.0001; Dunn’s, p<0.05; **Figure 4A**). In addition, Tg+GCV mice showed a decline in the weight of preputial glands which produce pheromones (WT ctr: 62.67 ± 4.28 mg; WT+GCV: 68.34 ± 5.35 mg; Tg ctr: 61.53 ± 3.13 mg; Tg+GCV: 47.83 ± 3.39 mg; Kruskal-Wallis, p=0.03; Dunn’s, p<0.05), whereas no change in the seminal gland weight was observed between the four groups (WT ctr: 227.2 ± 15.17 mg; WT+GCV: 226.4 ± 13.56 mg; Tg ctr: 254.3 ± 32.02 mg; Tg+GCV: 219.32 ± 4.5 mg; Kruskal-Wallis, p>0.05).

Following a human chorionic gonadotropin (hCG) injection, a stimulation that elicits the release of the total testosterone content from the testes (36), a significant decrease of about 50% of the mean plasma testosterone levels was observed in Tg+GCV compared to control groups (Kruskal-Wallis, p=0.04, Dunn’s, p<0.05; **Figure 4B**). Anatomical analysis of the testes sections showed that the three control groups, i.e. WT ctr (**Figure 4C**), WT+GCV (**Figure 4D**) and Tg ctr (**Figure 4E**), had normal seminiferous tubules. In contrast, the GCV treatment resulted in severe morphological alterations including vacuolization of seminiferous epithelium and no visible spermatozoa in the tube lumen in the Tg+GCV male mice (**Figure 4F**). Interestingly, the seminiferous tubules of Tg+GCV mice only contained Sertoli cells, spermatogonia and some spermatocytes. The stages downstream the spermatogonia stage were affected by the GCV treatment and no spermatids or spermatozoa could be observed in the tubules suggesting that in male Tg+GCV mice, spermatogenesis had stopped at the leptotene spermatocyte stage which is known to be highly sensitive to testosterone variations (44). Some of the seminiferous tubules also contained apoptotic vesicles corresponding to phagocytosis of spermatogonia by the Sertoli cells. The quantification of the different cell types in the seminiferous tubes uncovered that the Tg+GCV mice have significantly less spermatogonia and spermatocytes than the control mice (Kruskal-Wallis, p=0.0002; Dunn’s, p<0.05; **Supplementary Figure 3**). Moreover, no *Gfap* expression was detected in the testes of the control mice (data not shown) and the expression of the *tk* gene was undetectable in testes of Tg mice as shown by RT-PCR (**Supplementary Figure 4**), indicating that the anatomical defects observed in the testes were not due to a direct peripheral effect of GCV. These findings demonstrate that central GCV administration in Tg mice leads to a rapid and profound disruption of spermatogenesis and severe degradation of the testicular morphology. Furthermore, histochemical analysis of intestine sections reveals no alteration of the ileum with no trace of inflammation or haemorrhagic necrosis, in any of the mouse groups (**Supplementary Figures 5A–D**) as already reported in transgenic mice treated with peripheral administration of GCV (s.c.) for 11 days or more (33). This suggests that peripheral passage of the GCV through the blood-bain-barrier (BBB), if any, is too low to alter enteric glia.

Since testosterone release is triggered by the action of the pituitary hormone LH on Leydig cells, we then analysed circulating LH levels. Although there is a great variability because LH is secreted in a pulsatile and irregular manner, a significant decrease in the mean serum LH concentrations was observed in Tg+GCV male mice, (Kruskal-Wallis, p=0.04; Dunn’s, p<0.05; **Figure 4G**) compared to control groups except for WT+GCV mice. In contrast, no significant differences were found in the mean levels of FSH, which regulate Sertoli cell function between the four groups (Kruskal-Wallis, p>0.05; **Figure 4H**). Together these data strongly suggest that 4 weeks of i.c.v. GCV administration in the 3V results in severe hypogonadotropic hypogonadism in GFAP-Tk mice.

**Figure 5:**
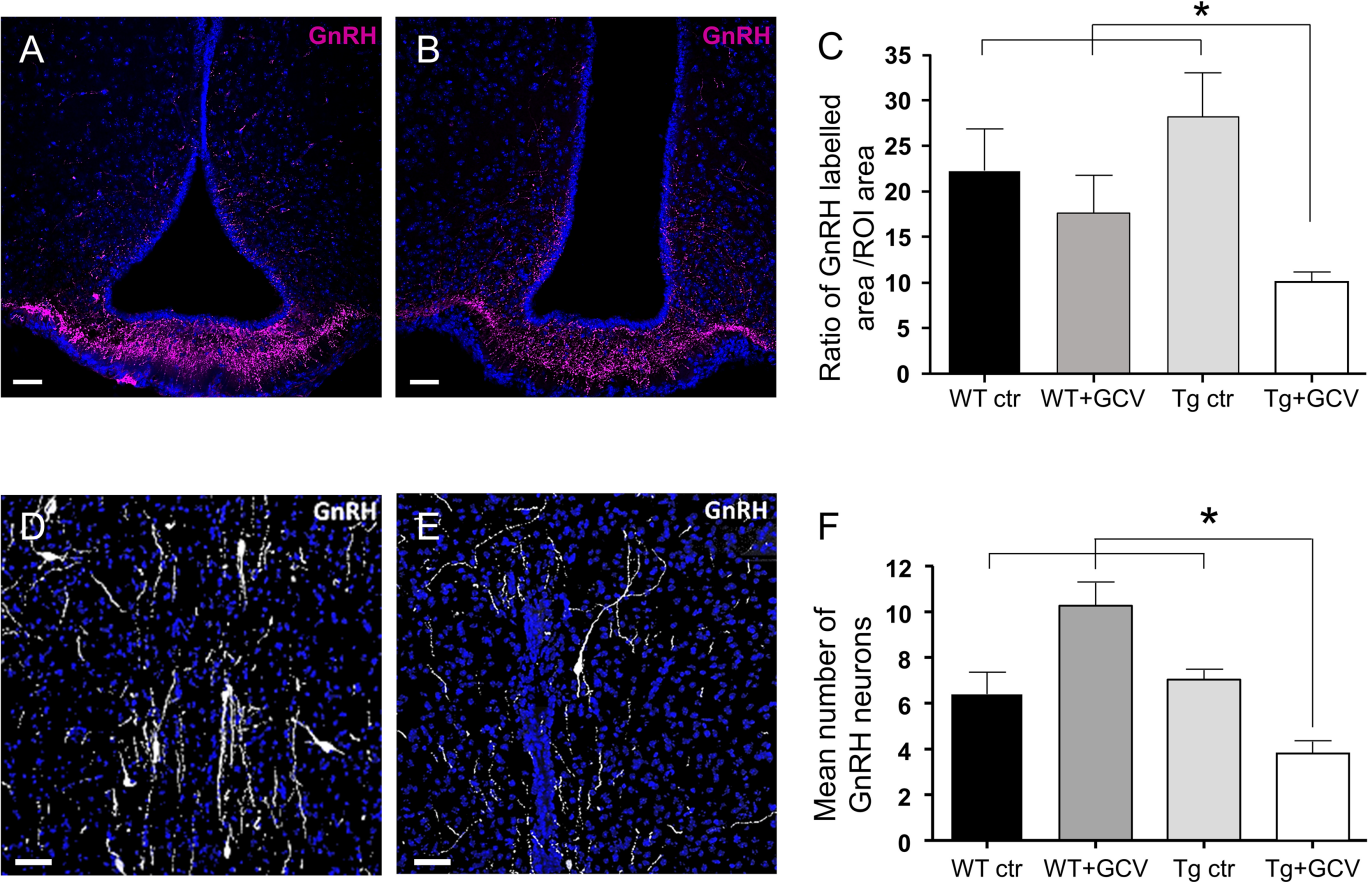
Effect of central GCV administration in Tg mice on GnRH immunoreactivity in the preoptic region and the median eminence. **(A, B)** Representative images of GnRH fibers in the median eminence of control **(A)** and Tg+GCV **(B)** male mice. Statistical analysis revealed a significantly decreased GnRH expression in Tg+GCV mice as compared to the three control groups **(C)**. **(D, E)** Representative images of GnRH neurons in the preoptic area (POA) of control **(D)** and Tg+GCV **(E)** male mice. **(F)** Mean number of GnRH neurons in the POA per slice, n≥3 slices per group. Statistical analysis revealed a significantly decreased GnRH neuron number in the POA in Tg+GCV male mice **(F)**. Nuclei coloration with Hoechst (blue). Data are expressed as the mean ± SEM. *p<0.05. Scale bars, 20 µm.

We next sought to investigate the consequences of GFAP-expressing tanycytic depletion on the GnRH system within the hypothalamus. Tanycytes of the ME tightly control the access of GnRH nerve terminals to the pituitary portal blood vessels (45, 46). Furthermore, tanycytes in the wall of the 3V have recently been shown to be able to modulate the activity of neurons in the AN (47), which is a key site for the feedback action of gonadal steroids and where Kisspeptin neurons reside. While neither Kisspeptin immunoreactivity nor the number of estradiol receptor alpha (ERα)-positive cells were affected by GCV treatment in the AN (Kruskal-Wallis, p>0.05; **Supplementary Figures 6A, B**), the density of the GnRH neuronal fibers was found to be significantly decreased in the ME of GCV-treated mice (Kruskal-Wallis, p=0.02, Dunn’s, p<0.05; **Figures 5A–C**). Surprisingly, this decreased density of GnRH neuronal fibers in the ME was associated with a 50% loss in the number of GnRH-immunoreactive neuronal cell bodies in the POA (Kruskal-Wallis, p=0.002, Dunn’s, p<0.05; **Figures 5D–F**). These results support the view that the morphofunctional interaction between GnRH axon terminals and tanycytes may not be only necessary for the control of GnRH release into the pituitary portal blood but also for GnRH expression and/or GnRH neuronal survival in the hypothalamus.

### GFAP-Expressing Tanycyte Depletion Alters Male Sexual Behavior

**Figure 6:**
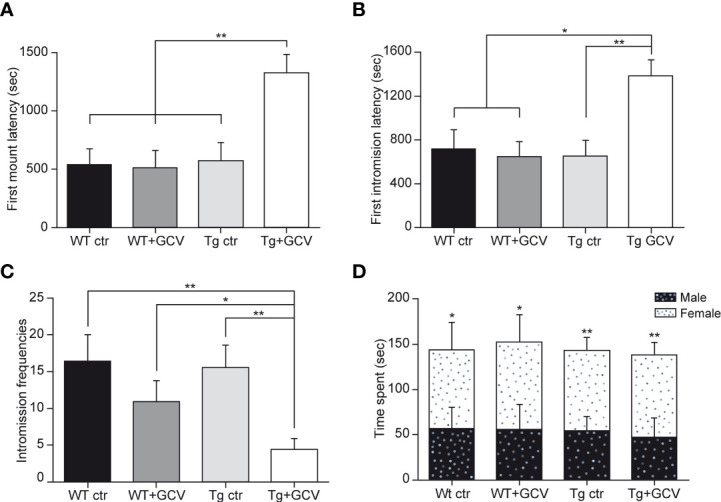
Central GCV administration in Tg mice affected male sexual behavior. The latencies to first mount **(A)** and to first intromission **(B)** are increased in Tg+GCV male mice, while the intromission frequencies **(C)** are decreased after central GCV administration in Tg mice. Males of the four groups spend more time in close contact to a receptive female than an unfamiliar male **(D)**. Data are expressed as the mean ± SEM, n≥6 for each group. *p<0.05, **p<0.01.

To further explore the consequences of gonadotropic axis dysregulation by GFAP-expressing tanycyte depletion, two main well-established paradigms of sexual behavior in male mice, namely the latency to the first mount and intromission and the frequency of intromissions were analysed. To achieve this, male mice of the four experimental groups were exposed to receptive females for 30 minutes. In this test, the Tg+GCV male mice exhibited increased latencies to the first mount (Kruskal-Wallis, p=0.01; Dunn’s, p<0.05; **Figure 6A**) as well as for the first intromission (Kruskal-Wallis, p=0.03 Dunn’s, p<0.05; **Figure 6B**) together with a lower frequency of intromissions (Kruskal-Wallis, p=0.008; Mann-Whitney, p<0.05; **Figure 6C**). To test whether the decrease in sexual performance of the Tg+GCV male mice was due to a lack of sexual attraction towards females, we performed a sexual preference test by exposing male mice of the four groups to both an unfamiliar male and a receptive female. Males from all groups spent more time near the female than near the unfamiliar male (Wilcoxon, WT ctr p=0.05, WT+GCV p=0.03, Tg ctr p=0.001 and Tg+GCV p=0.004; **Figure 6D**), indicating that GCV treatment did not alter the sexual preference of the treated males for females. Altogether these results show that GCV-treatment leads to an impairment of the copulative behavior without affecting sexual preference in male mice.

### Central GCV Administration in Tg Mice Does Not Alter Anxiety Levels

The hypothalamus regulates stress and anxiety responses *via* the corticotropic axis and tanycytes are also known to be morphologically associated with corticotropin releasing hormone (CRH) axon terminals. To test whether ablating GFAP-positive tanycytes might also lead to depressive/anxiety behavior, two behavioral studies were conducted, namely the elevated plus maze and the marble burying tests. The GCV treatment did not modify the number of entries into each zone of the elevated plus maze (two-way ANOVA, p>0.05; **Supplementary Figure 7A**) and Tg+GCV male mice spent as much time in each area of the elevated plus maze device as the three control groups (two-way ANOVA, p>0.05; **Supplementary Figure 7B**). The marble burying test, an anxiety test that partly depends on hippocampal function (48) showed that animals buried the same number of marbles whichever group they belonged to (Kruskal-Wallis, p>0.05; **Supplementary Figure 7C**). Additionally, no significant difference was detected in the mean plasma cortisol concentrations between the four groups (Kruskal-Wallis, p>0.05; **Supplementary Figure 7D**), consistent with the findings that the GCV treatment had no effect on the anxiety level in transgenic mice.

## Discussion

Dividing GFAP-positive adult cells constitute the unique pool of NSCs in the SVZ and SGZ (1–6) and their ablation results in adult neurogenesis depletion within the two canonical niches (2, 4). In the hypothalamus, tanycytes have been described as the NSCs capable of generating neurons and glial cells (8, 49). Despite strong morphological similarities between the four different populations of tanycytes, recent single-cell RNA sequencing (scRNA-Seq) studies showed much greater molecular diversity than previously thought among tanycytes (31, 50). Although all tanycytic populations express Sox2, vimentin and Nestin, only the α-tanycyte subtypes express GFAP (25, 31), while Fgf10 (fibroblast growth factor 10) and BLBP (Brain Lipid-Binding Protein) are only expressed by the β subtypes (25, 51, 52), suggesting distinct roles of these two NSPC populations. In the current study, we used a Tk-transgenic mouse line in which only dividing GFAP-positive NSPCs are selectively removed while non-dividing GFAP-positive cells are spared by ganciclovir treatment (2).

Hypothalamic dividing GFAP-positive NSPCs, that were selectively targeted, correspond mainly to one of the mitogenic regions identified in the tanycytic layer lining the 3V wall, namely the α1 plus a dorsal subset of α2 tanycytes (25). The *in vitro* depletion of hypothalamic dividing GFAP-expressing cells markedly decreased the neurospherogenic capacities of this region, indicating that elimination of α-tanycytes has almost completely halted cell proliferation. Interestingly, knowing that the entire MBH has been cultivated, the 90% decrease in the number of neurospheres and the 80% decrease in their size following GCV treatment, strongly suggest that β-tanycytes do not constitute the main pool of hypothalamic NSPCs. *In vivo*, the depletion of dividing GFAP-expressing tanycytes resulted in a reduction of the NSPC marker expression including vimentin and Sox2 not only in the VMH but also in the AN and ME. This result suggests a close relationship between α- and β-tanycytes. In agreement with these results, α-tanycytes expressing both GFAP and Sox2 are capable to self-renew, generate neurons and astrocytes and give rise to β-tanycytes (14, 25, 53). Together, these results support the assumption that GFAP-expressing α-tanycytes exhibit marked neurospherogenic capacities *in vitro* (14, 25, 53), constitute the main source of NSPCs, similar to those of the two canonical niches (2), and are likely to play a crucial role in the generation of β-tanycytes.

Several studies have reported that newborn cells, most likely generated from tanycytes, integrate the hypothalamic neuronal networks of the AN and ME and are engaged in the regulation of energy homeostasis (12, 23, 24, 52). A decrease in hypothalamic new cell generation induces obesity and the development of metabolic disorders associated with insulin resistance (24). In turn, obesity induced by a high fat diet was shown to impair hypothalamic neurogenesis and disrupt energy balance (24, 49). In addition, a 2-months high fat diet induces a significant increase in the proliferation of β-tanycytes and the production of new cells in the ME (23). In order to determine whether hypothalamic GFAP-expressing tanycytes are implicated in metabolism regulation, we explored the effect of their depletion on body weight and food intake in transgenic male mice. The current study demonstrates that the suppression of GFAP-expressing tanycytes had no effect on both parameters, nor on the number of anorexigenic and orexigenic neurons i.e. POMC and NPY neurons respectively. Two hypotheses could explain these results. First, this targeted population of NSPCs, corresponding mainly to the α1 and dorsal α2 tanycytes, would not be directly involved in the regulation of food intake, unlike the β tanycytes (23, 24). Alternatively, no effect was detected because the animals were unchallenged. The exploration of whether a diet challenge could affect food intake in GFAP-expressing tanycyte-depleted mice might bring insight to this question. Indeed, metabolic consequences at a longer term cannot be excluded, as previously demonstrated by the alteration of body weight and food intake 3 months and 10 months respectively after induced inflammation in hypothalamic NSCs (24).

We then investigated the involvement of hypothalamic GFAP-expressing tanycytes in the control of reproduction, a function also orchestrated by the hypothalamus. In a recent study in sheep, a seasonal mammal, we showed that administration of the antimitotic drug AraC in the 3V downregulates the production of hypothalamic new neurons identified by the expression of doublecortin and alters the timing of reproduction (54). In an aged mouse model, GnRH was shown to stimulate the hypothalamic mitogenic activity (55). These data suggest a strong link between the hypothalamic neural stem cell niche and the neural circuits controlling reproduction. The present study shows that the sexual behavior and sexual performance of male Tg+GCV mice are strongly altered after 4 weeks of GCV treatment and this without affecting the neural circuits involved in sexual partner recognition. This phenotype does not result from a higher level of anxiety in Tg+GCV mice as they exhibit plasma cortisol concentrations and levels of anxiety comparable to those in the control groups.

A thorough analysis of the reproductive system of the Tg+GCV male mice showed a significant decrease in testes weight and a drastic reduction in testosterone secretion by the Leydig’s cells. In turn, the drop in testosterone secretion triggered the vacuolization of the seminiferous tubules probably due to the cessation of spermatogenesis and severe hypogonadism. These reduced levels of testosterone are also likely the cause of the strong alteration in sexual behavior seen in Tg+GCV males. Several studies have reported that prenatal or adult exposure to GCV can be spermatotoxic and induce drastic effects on testes (56, 57) even if a recent systematic overview indicates a limited number of studies investigating the effect of GCV and could not conclude on the spermatotoxic effects of this substance (58). In all cases, effects of GCV on testes and germ lines were found when high doses were administered subcutaneously (100 – 300 mg/kg) (56, 57). In the present study, the doses used for *i.c.v*. administrations of GCV were much lower (1.34 µg/kg/day) than that those used subcutaneously. However, a weak passage of GCV through the BBB reaching the periphery could still be possible. To further confirm the absence of peripheral effect, histopathological analysis of gastrointestinal tract was performed since i.p. GCV treated mice exhibited severe inflammation and necrosis of the jejunum and ileum (33). No alteration of the intestine was evidenced in any of the two groups of GCV-treated mice. In addition, in case of leakage, WT+GCV mice would have had the same phenotype as Tg+GCV, ones which is not the case. Overall, these data indicate that there is no peripheral leakage of GCV, or if so, the dose is so low that the morphology of intestine is not altered, suggesting that any peripheral effect is highly unlikely.

Testosterone inhibits GnRH/LH secretion *via* negative feedback onto the hypothalamus. The drop in testosterone levels being associated with decreased circulating LH levels in Tg+GCV mice strongly suggests that the hypogonadism in these mice is due to a central defect. In agreement, we found that GCV-mediated depletion in tanycytes results in an alteration of the GnRH immunoreactivity, which is markedly decreased both at the GnRH neuronal cell bodies in the POA and the termination field of GnRH neurons in the ME. Interestingly, GCV treatment in transgenic mice did not affect FSH levels. These results are consistent with previous studies in which the use of GnRH antagonists in humans induce an immediate decrease in LH and testosterone secretions, while the decrease in FSH is delayed and weaker because of its short half-life (59, 60). These antagonist treatments cause a rapid hypogonadism phenotype that is dependent on fluctuations in pituitary hormones and testosterone, comparable to our data (59, 60). In ewes (61), non-human primates (62), and ovariectomized rats (63, 64), the administration of a GnRH antagonist as well as the hypothalamic-pituitary disconnection (65) suppressed pulsatile LH secretion but had a minimal effect on FSH secretion, indicating that GnRH differentially controls the secretion of LH and FSH. However, a GCV treatment of more than 4 weeks of Tg mice might also have altered FSH secretion. Moreover, the administration of AraC in the lateral ventricles of female rats alters the neural circuitry that controls adult GnRH/LH release and decreases the preovulatory LH surge (66). Altogether, these data point to a role for the adult hypothalamic neural stem cell niche in the control of the reproductive function.

The cellular/molecular mechanisms inducing GnRH deficiency following suppression of hypothalamic GFAP-expressing tanycytes are not known. Kisspeptin-54 being the most potent secretagogue of the GnRH system (67), and having been reported to induce a reduction in testicular weight as well as testicular degeneration in adult male rats when chronically administrated (68), was one of the candidates. However, Kisspeptin expression in fibers located in the AN is not altered in GCV-treated transgenic mice. Similarly, the expression of gonadal steroid receptors such as the estrogen receptor-α (ERα) appeared to be unaffected. Alternately, a more direct hypothesis involves the β-tanycytes located in the ME, which mediate the release of GnRH into the pituitary portal blood vessels by controlling the direct access of GnRH nerve endings to the vascular wall but also by setting up specific communication channels with GnRH nerve terminals and supporting neuronal survival (69, 70). However, this view is somewhat contradicted by a recent study reporting the physiological consequences of selective β-tanycyte ablation (71). Although it shows, as in the present study, that β-tanycyte ablation has no effect on food intake and body weight, the authors did not observe any significant changes in serum levels of LH and FSH. This discrepancy could be explained by the fact that the animals in this study were fed a diet containing tamoxifen for 3-weeks; tamoxifen being a potent modulator of gonadal steroid receptors leading to confounding effects on the activity of the gonadotropic axis. In particular, tamoxifen has been clinically shown to increase androgen levels and sperm concentration in males with idiopathic oligozoospermia (72).

GFAP-expressing α1- and dorsal α2-tanycytes may communicate with GnRH fibers traveling towards the ME, but do not interact morphologically with GnRH nerve terminals in the ME. However, their depletion caused a marked alteration in β-tanycyte number and/or morphology as evidenced by the significant decrease in vimentin and Sox2 labelling 4 weeks after GCV treatment (**Figures 3E–G**). These results are in agreement with the finding that α-tanycytes expressing GFAP self-renew and give rise to β-tanycytes (14, 25, 53, 72). The immunohistological changes observed in our study likely resulting in significant structural alterations, might hamper the tanycyte-to-GnRH-neuron communication processes controlling reproduction [see for review (73)]. Hence, one is tempted to speculate that this alteration in the structure and function of hypothalamic tanycytes may also account, at least in part, for the loss of GnRH immunoreactivity in the ME with possible consequences for overall expression of GnRH in the cell bodies or for the survival of GnRH neurons in the preoptic region. This phenomenon, which could be due to an alteration of the ability of tanycytes to fight against systemic inflammation at the blood-cerebrospinal barriers (24, 55, 74), affecting fitness and, maybe, viability of neuroendocrine systems (75) or promoting changes in gene-miRNA micronetworks (76, 77) remains to be explored. As an alternate hypothesis, since tanycytes are able to form self-renewing neurospheres and produce primarily astrocytes (25), removal of GFAP-expressing tanycytes may have modified the glial microenvironment around the GnRH neurons and fibers. The microenvironment of GnRH neurons participates in the modulation of their activity in particular in the synthesis and secretion of GnRH. In the rat POA, astrocytes interact closely with cell bodies and GnRH neuronal processes, in adult (78) and infancy (10). In addition, glial cells synthesize and release prostaglandin E2 (PGE2) known to enhance the electrical activity of GnRH neurons and therefore its pulsatile secretion (79). Future research examining the glial microenvironment surrounding the GnRH neurons and fibers and recordings of GnRH neuronal activity would shed light on mechanisms leading to the loss of GnRH immunoreactivity in Tg+GCV mice.

In conclusion, our results established the importance of α-tanycytes in the production and maintenance of the NSPC pool of the entire MBH. In particular, they show that the elimination of GFAP-expressing tanycytes severely impairs the activity and function of GnRH neurons leading to hypogonadotropic hypogonadism and alters sexual behaviors in male mice, highlighting their key role in the control of reproduction.

## Data Availability Statement

The raw data supporting the conclusions of this article will be made available by the authors, without undue reservation.

## Ethics Statement

The animal study was reviewed and approved by The Val de Loire animal experimentation ethics committee (CEEAVdL).

## Author Contributions

All authors contributed to data collection, analysis and interpretation, or drafting and revising the manuscript, and approved the final manuscript for publication. Specifically, LB, MB, and MM designed the study. LB, MB, DP, M-LC, and VL contributed to data collection. LB, MB, AS, VP, PV, DP, and MM contributed to data analysis. LB, MB, AS, VP, DP, and MM contributed to data interpretation and preparation of the manuscript. All authors contributed to the article and approved the submitted version.

## Funding

LB received a grant from the Région Centre. This work was funded by the PHASE department of INRAE. This project was funded by the Agence Nationale de la Recherche ANR-16-CE37-0006 (to MM) and the European Research Council (ERC) Synergy Grant WATCH No 810331 (to VP).

## Conflict of Interest

The authors declare that the research was conducted in the absence of any commercial or financial relationships that could be construed as a potential conflict of interest.

## Publisher’s Note

All claims expressed in this article are solely those of the authors and do not necessarily represent those of their affiliated organizations, or those of the publisher, the editors and the reviewers. Any product that may be evaluated in this article, or claim that may be made by its manufacturer, is not guaranteed or endorsed by the publisher.
