# Supplementary material for: Selective Depletion of Adult GFAP-Expressing Tanycytes Leads to Hypogonadotropic Hypogonadism in Males
Source: Front Endocrinol (Lausanne). 2022 Mar 16;13:869019. doi: 10.3389/fendo.2022.869019 (PMC8966543; doi:10.3389/fendo.2022.869019)
Supplement: Supplementary file 1 [file Presentation_1.pptx]

## Slide 1
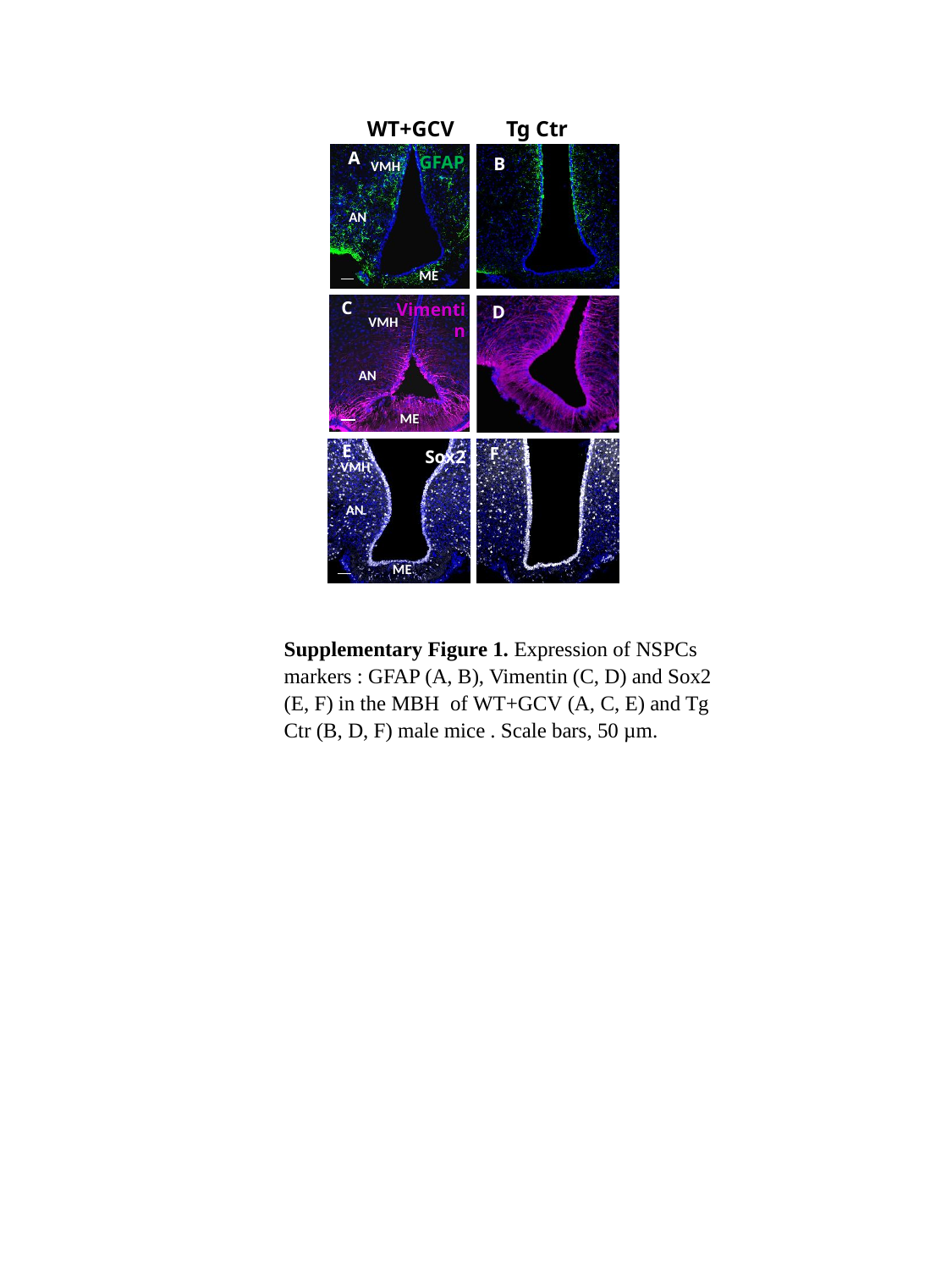

Tg Ctr
WT+GCV
A
GFAP
B
VMH
AN
ME
C
Vimentin
D
VMH
AN
ME
E
F
Sox2
VMH
AN
ME
Supplementary Figure 1. Expression of NSPCs markers : GFAP (A, B), Vimentin (C, D) and Sox2 (E, F) in the MBH of WT+GCV (A, C, E) and Tg Ctr (B, D, F) male mice . Scale bars, 50 µm.
E
F

## Slide 2
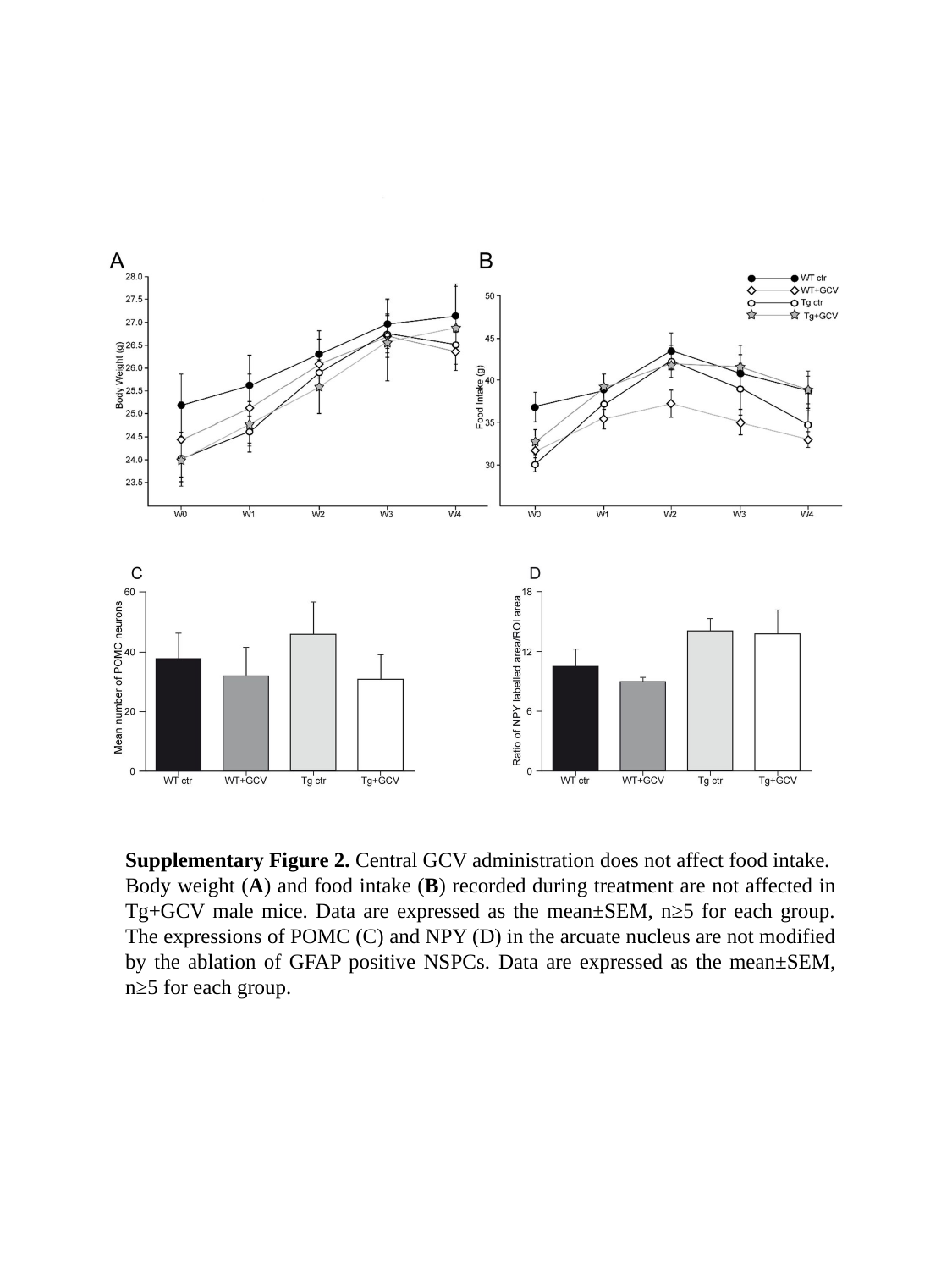

Supplementary Figure 2. Central GCV administration does not affect food intake.
Body weight (A) and food intake (B) recorded during treatment are not affected in Tg+GCV male mice. Data are expressed as the mean±SEM, n≥5 for each group. The expressions of POMC (C) and NPY (D) in the arcuate nucleus are not modified by the ablation of GFAP positive NSPCs. Data are expressed as the mean±SEM, n≥5 for each group.

## Slide 3
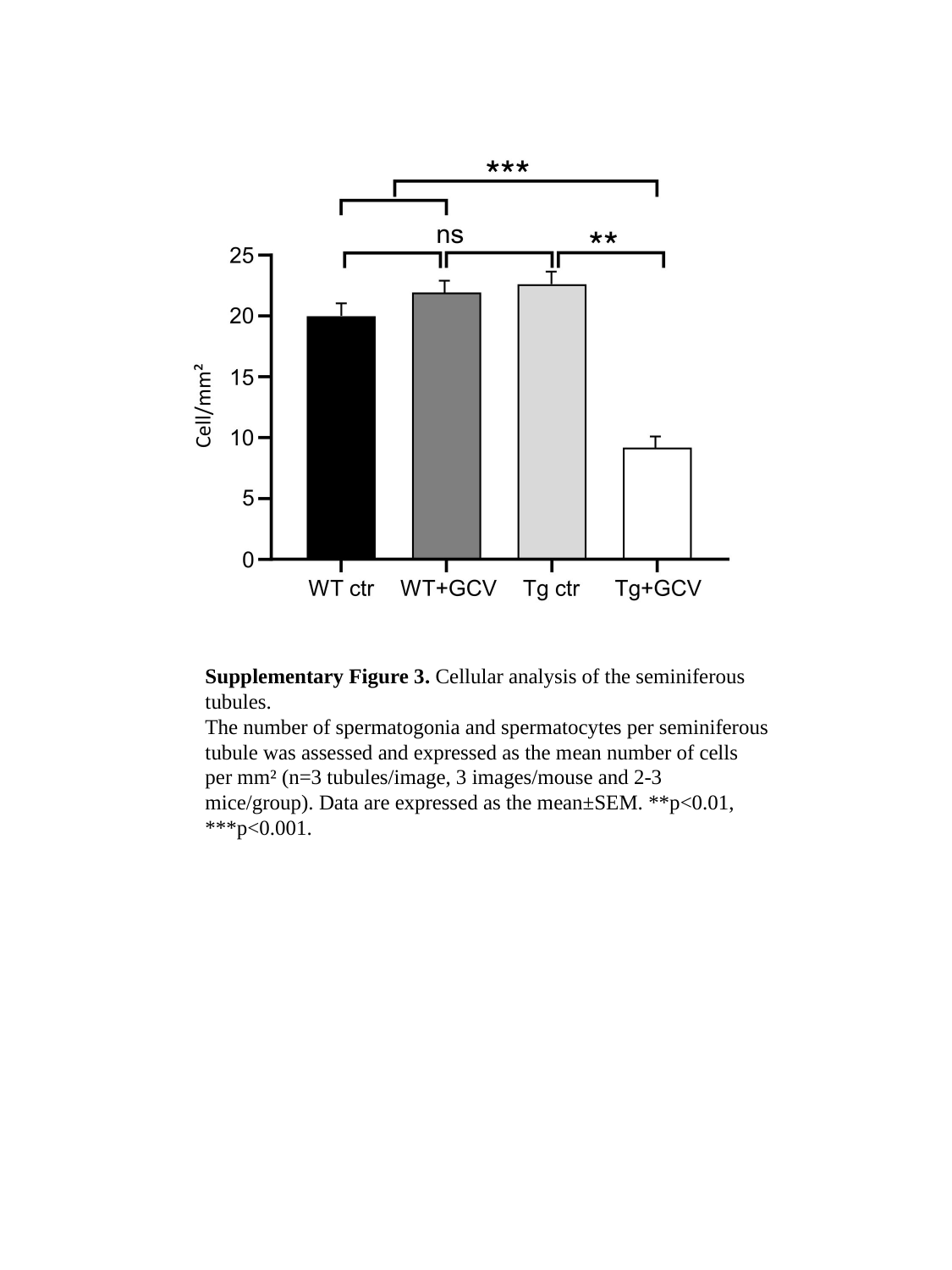

Supplementary Figure 3. Cellular analysis of the seminiferous tubules.
The number of spermatogonia and spermatocytes per seminiferous tubule was assessed and expressed as the mean number of cells per mm² (n=3 tubules/image, 3 images/mouse and 2-3 mice/group). Data are expressed as the mean±SEM. **p<0.01, ***p<0.001.

## Slide 4
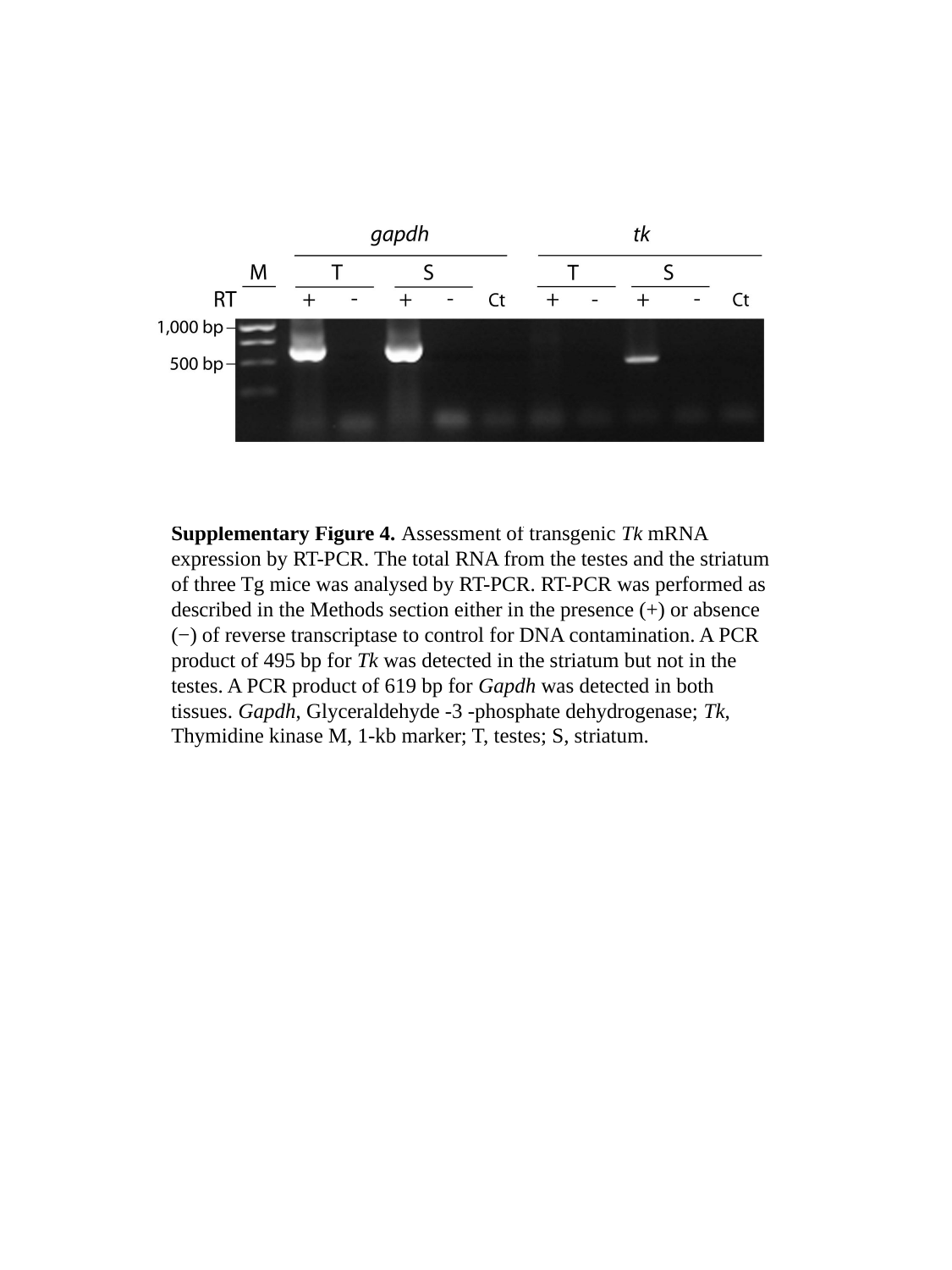

Supplementary Figure 4. Assessment of transgenic Tk mRNA expression by RT-PCR. The total RNA from the testes and the striatum of three Tg mice was analysed by RT-PCR. RT-PCR was performed as described in the Methods section either in the presence (+) or absence (−) of reverse transcriptase to control for DNA contamination. A PCR product of 495 bp for Tk was detected in the striatum but not in the testes. A PCR product of 619 bp for Gapdh was detected in both tissues. Gapdh, Glyceraldehyde -3 -phosphate dehydrogenase; Tk, Thymidine kinase M, 1-kb marker; T, testes; S, striatum.

## Slide 5
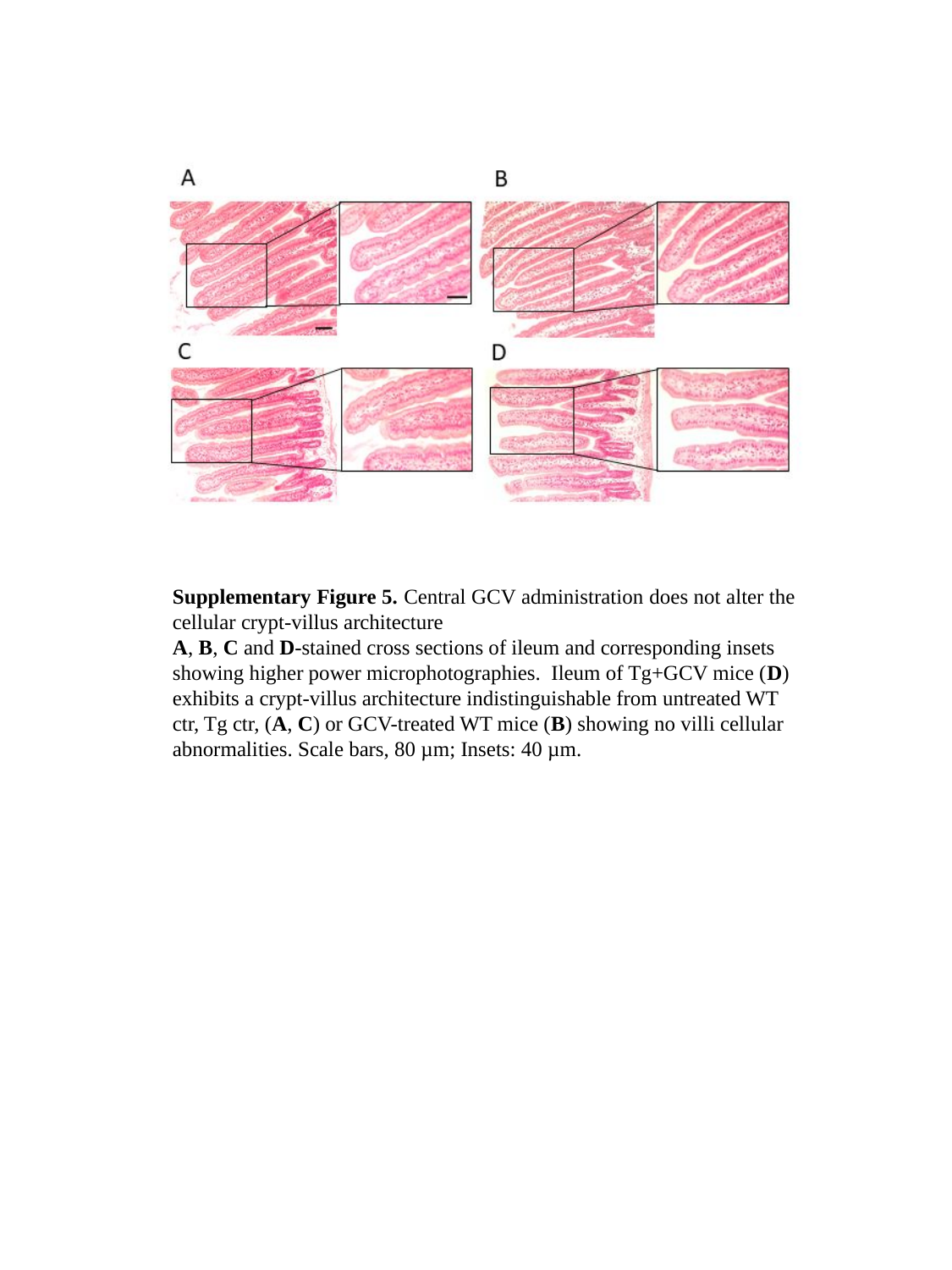

Supplementary Figure 5. Central GCV administration does not alter the cellular crypt-villus architecture
A, B, C and D-stained cross sections of ileum and corresponding insets showing higher power microphotographies. Ileum of Tg+GCV mice (D) exhibits a crypt-villus architecture indistinguishable from untreated WT ctr, Tg ctr, (A, C) or GCV-treated WT mice (B) showing no villi cellular abnormalities. Scale bars, 80 µm; Insets: 40 µm.

## Slide 6
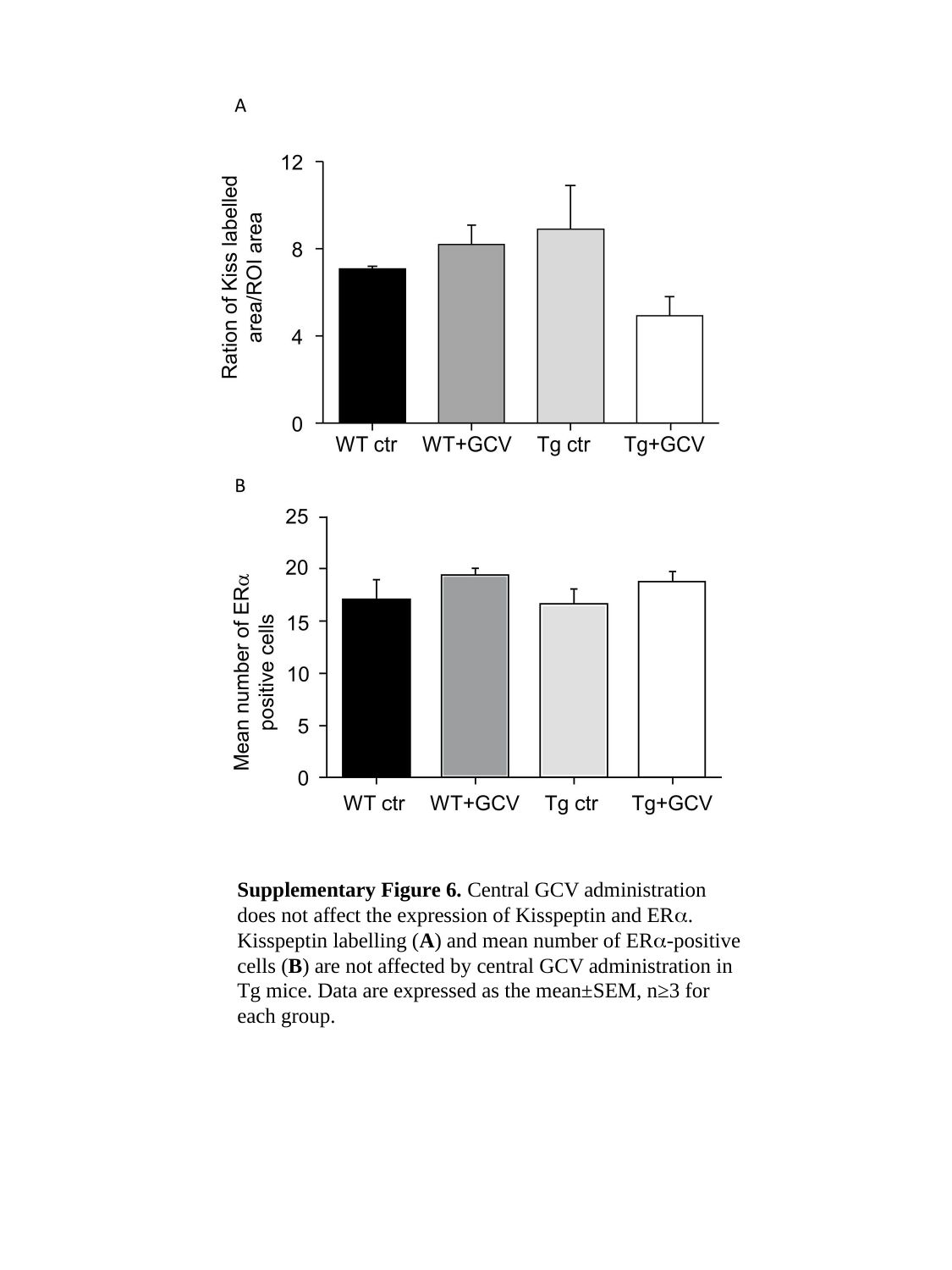

Supplementary Figure 6. Central GCV administration does not affect the expression of Kisspeptin and ERα.
Kisspeptin labelling (A) and mean number of ERα-positive cells (B) are not affected by central GCV administration in Tg mice. Data are expressed as the mean±SEM, n≥3 for each group.

## Slide 7
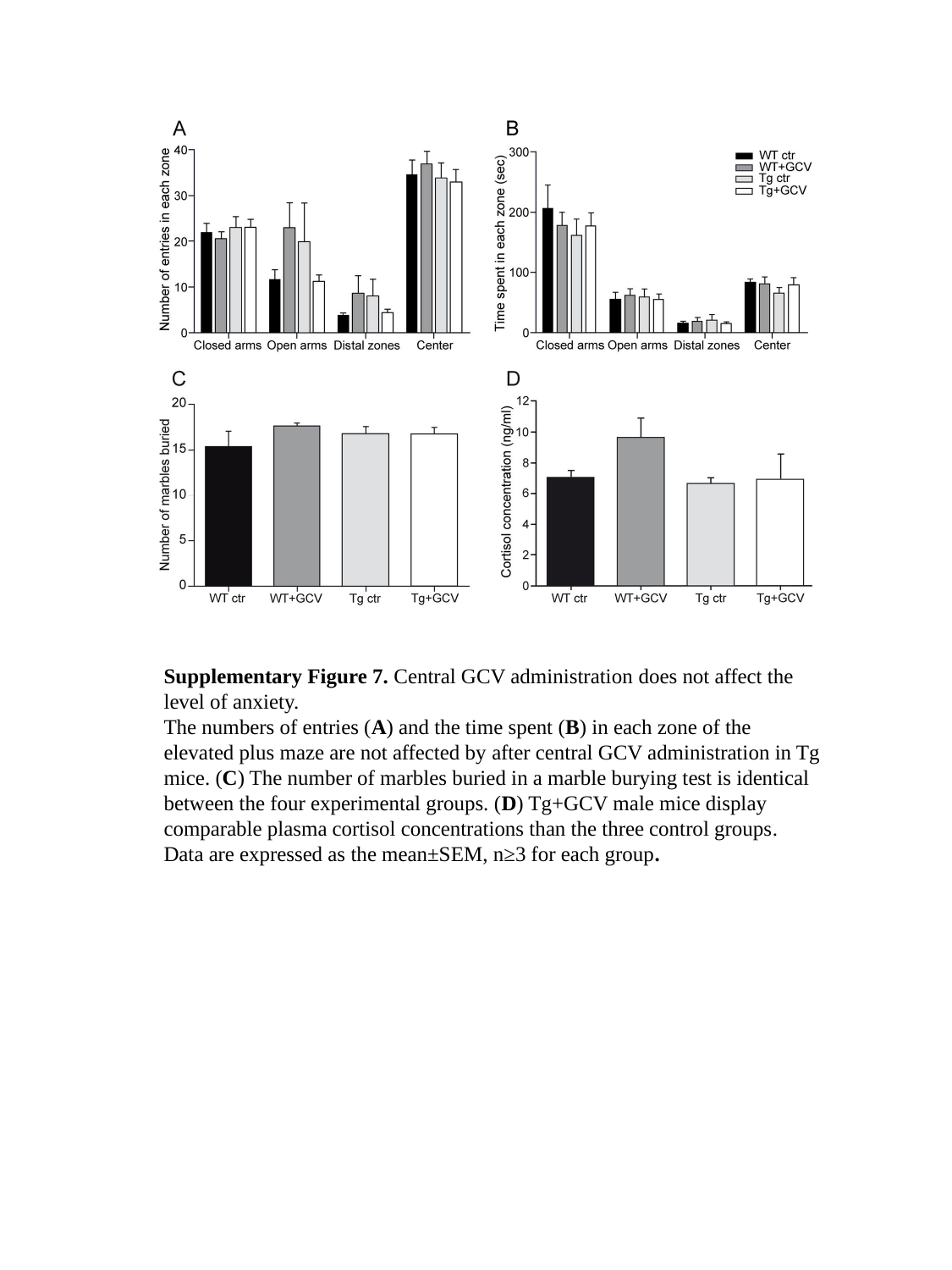

Supplementary Figure 7. Central GCV administration does not affect the level of anxiety.
The numbers of entries (A) and the time spent (B) in each zone of the elevated plus maze are not affected by after central GCV administration in Tg mice. (C) The number of marbles buried in a marble burying test is identical between the four experimental groups. (D) Tg+GCV male mice display comparable plasma cortisol concentrations than the three control groups. Data are expressed as the mean±SEM, n≥3 for each group.
